# Polymerizable Cholinium-Based Antibiotics for Polymer Carriers: Systems with Combined Load of Cloxacillin and Ampicillin

**DOI:** 10.3390/molecules29245973

**Published:** 2024-12-18

**Authors:** Shadi Keihankhadiv, Dorota Neugebauer

**Affiliations:** Department of Physical Chemistry and Technology of Polymers, Faculty of Chemistry, Silesian University of Technology, 44-100 Gliwice, Poland; shadi.keihankhadiv@polsl.pl

**Keywords:** linear polymers, cloxacillin, ampicillin, ionic liquid, choline, drug delivery system

## Abstract

Single and dual-drug delivery systems (DDSs) based on linear choline polymers were designed through the controlled polymerization of a pharmaceutically functionalized monomer, i.e., [2-(methacryloyloxy)ethyl]trimethylammonium, with counterions of cloxacillin (TMAMA/CLX), or its copolymerization with [2-(methacryloyloxy)ethyl]trimethylammonium with ampicillin (TMAMA/AMP), providing antibiotic properties. This strategy was effective in attaining well-defined linear copolymers with 38–93 mol. % of TMAMA content, which were regulated by the initial ratio of TMAMA to methyl methacrylate comonomer. The polymer compositions were controlled by the total monomer conversion (40–75%), resulting in a variable degree of polymerization (DP_n_ = 160–300) and pharmaceutical anion contents (CLX^−^ 51–80% and AMP^−^ 78–87%). In aqueous solution, the polymers formed particles with sizes ranging between 274 and 380 nm for CLX^−^ systems and 288–348 nm for CLX^−^/AMP^−^ systems. In vitro drug release, driven by the exchange of pharmaceutical anions with phosphate ions in phosphate-buffered saline (PBS), imitating a physiological fluid, demonstrated release efficiencies of 58–76% for CLX^−^ (10.5–13.6 µg/mL) in single systems, and 91–100% for CLX^−^ (12.9–15.1 µg/mL) and 97–100% for AMP^−^ (21.1–23.3 µg/mL) in dual systems. Compared to conventional systems delivering antibiotics without a polymer carrier, the choline-based polymer DDS attained satisfactory levels of drug loading content and (co-)release from the polymer carriers, offering a promising alternative for antibiotic delivery.

## 1. Introduction

Choline, identified as 2-hydroxyethyl trimethylammonium chloride, is naturally synthesized in the human liver and is found in phospholipids such as phosphatidylcholine or lecithin. This organic salt is classified as an ionic liquid (IL) [1], known for its chemical stability, ability to enhance solubility, and modifiability through ion exchange to adjust physical and chemical properties [2,3,4]. Many ILs exhibit nontoxicity and biocompatibility, making them suitable for biomedical applications. Their biological properties include enhancing skin penetration [5], acting as antibacterial agents [6], and functioning as stabilizers [7,8]. They also demonstrate cytotoxicity, local anesthetic effects, anti-fungal and anti-acne activities, and antibiotic properties [3,9,10]. Furthermore, the versatility of ILs extends to their ability to accommodate a wide range of pharmaceutical substances, including antiviral and antimicrobial agents, antioxidants, anticoagulants, nonsteroidal anti-inflammatory drugs, anticancer drugs, ophthalmic drugs, and other drugs [3,11,12,13,14,15,16]. For instance, betaine- and L-carnitine-based ILs have been reported to be solubilizing and stabilizing agents for bioactive diacerein in eye drops [17]. The widely utilized cholinium cation is particularly notable for its biodegradability, water-solubility, and low cost, making it suitable for various applications [18,19,20]. Its properties have been studied in combination with various bioactive compounds, including phenytoin [21], ampicillin (AMP) [22], nalidixic acid, niflumic acid, *p*-aminosalicylic acid, pyrazinoic acid, and picolinic acid [19]. These systems have shown improved solubility of active pharmaceutical ingredients, enhancing their ability to permeate the cell membrane [18].

In recent decades, the synthesis of precisely designed polymers with tailored architectures, compositions, chain homogeneity, and site-specific functionality [23,24,25], as well as desirable physicochemical and biochemical properties (e.g., mechanical strength, softness, self-healing, processability, tissue adhesiveness, bioactivity, and optional biodegradability) [26,27,28,29,30], has emerged as a powerful tool for developing versatile nanostructures applicable in biology and medicine. Tailored-made polymers synthesized using controlled polymerization methods have significantly advanced drug delivery systems (DDSs), offering both linear and branched polymer carriers with biotherapeutic functions [31,32,33,34]. Polymers conjugated with drugs or encapsulating drugs have been intensively studied to address challenges related to drug hydrophilicity [35,36]. Moreover, the co-delivery of multiple bioactive compounds has been explored to enhance primary drugs’ efficacy. The combination of conventional pharmacotherapies, including antibiotics, demonstrates improved antibacterial effectiveness and reduced drug resistance, while polymer carriers enhance the controlled pharmacodynamics and pharmacokinetics of DDSs [37,38,39].

The commercial choline ester derivative, [2-(methacryloyloxy)ethyl]trimethylammonium chloride, commonly referred to as methacroylcholine (TMAMA/Cl), functions as a choline-based IL and serves as the monomer for synthesizing polymerized ionic liquid (PIL) [40]. This PIL has been reported to deliver pharmaceutical anions through anion exchange within the polymer matrix [41,42] or to encapsulate various bioactive compounds [43,44,45,46,47], creating pharmaceutically active polymeric systems. Pharmaceutically active choline-based PILs have also been designed by polymerizing pharmaceutically functionalized choline monomers carrying salicylate [40], *p*-aminosalicylate [48], or fusidate [49] counterions. Additionally, graft copolymers have been synthesized using a combination of choline monomers containing fusidate and cloxacillin (CLX) [49], while other dual-drug systems have been synthesized with both *p*-aminosalicylate- and AMP-functionalized choline comonomers [50]. 

**Figure 1:**
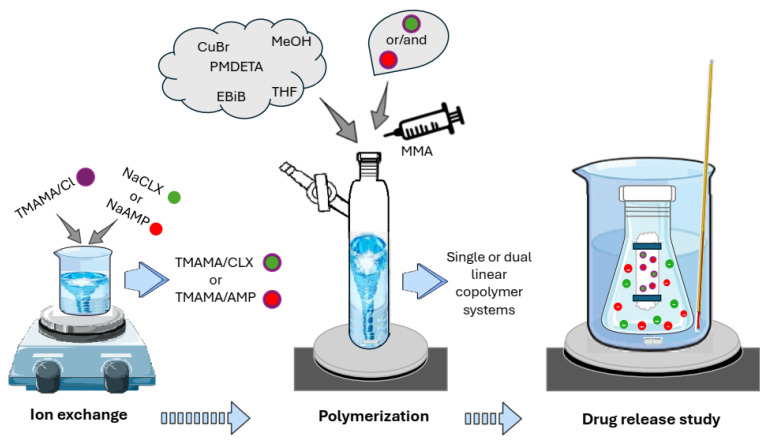
Schematic route from the modification of choline IL by pharmaceutical CLX and AMP anions to linear polymers as single and dual systems, including drug release in PBS at 37 °C.

In this research, we investigated linear polymers based on monomeric choline ionic liquid with a CLX anion (TMAMA/CLX), optionally copolymerized with an AMP-based choline methacrylate monomer (TMAMA/AMP). These well-defined copolymers were designed as either a single DDS carrying CLX^−^ or a dual DDS with CLX^−^ and AMP^−^ (Figure 1), where the drug anion is ionically bonded to the polymer matrix. Both drugs are antibiotics derived from semi-synthetic penicillin, demonstrating antimicrobial efficacy due to the presence of a beta-lactam ring [51,52]. They are effective against both Gram-positive and certain Gram-negative microorganisms [53] and are widely used to treat bacterial infections affecting the ears, nose, throat, bones, and lungs and post-operative wounds [54]. The commercially available combination of AMP and CLX is marketed under various brand names, such as Ampiclox and Cloxam, typically in capsule or oral suspension forms without polymer carriers. This makes both antibiotics suitable for use in either individual treatment or combined therapies. The synthesized polymer systems were characterized to evaluate their effectiveness in drug delivery through the evaluation of drug content and a (co-)release in vitro study in physiological solution via anion exchange with phosphate ions, offering a controlled delivery mechanism. In comparison to previous literature reports for systems delivering AMP and CLX, the advantage of this novel DDS lies in its design, with the controlled radical polymerization of antibiotics incorporated in choline monomers to attain ionic polymers with regulated compositions and drug contents, as well as the option of AMP/CLX co-release by polymer carriers instead of separate delivery by various systems.

## 2. Results and Discussion

### 2.1. Monomeric Ionic Liquid Functionalized by Pharmaceutical Anions

**Figure 2:**
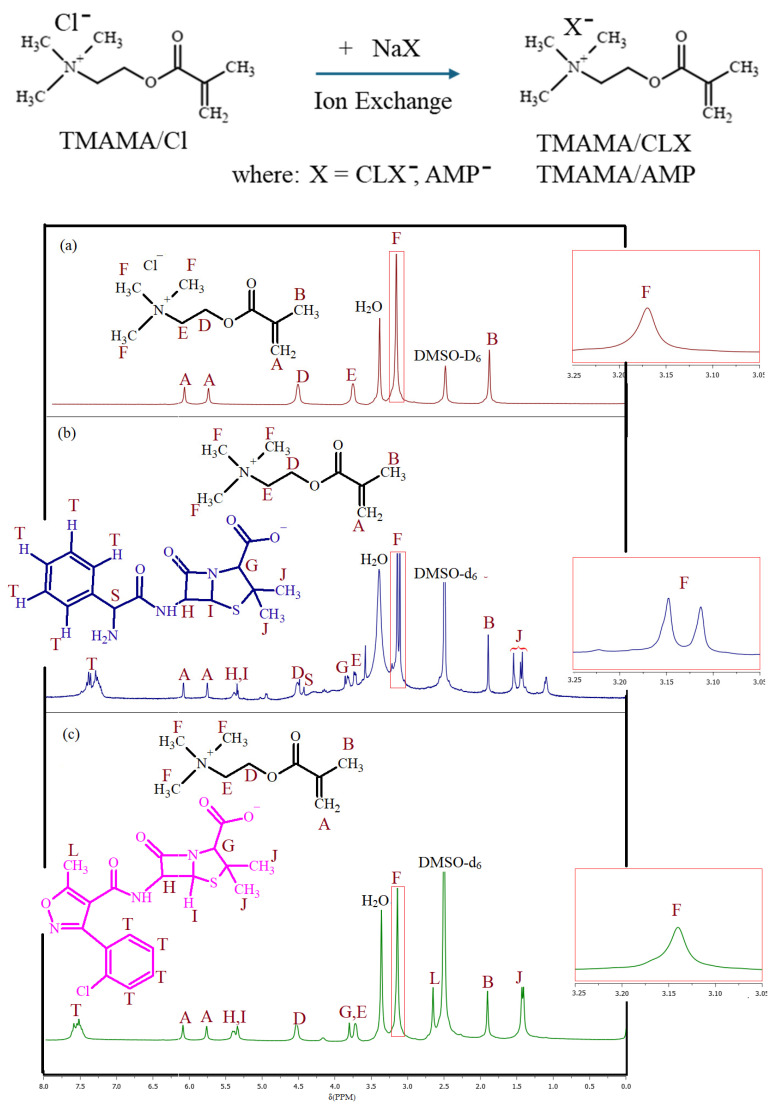
Ionic exchange of TMAMA/Cl with sodium salts of CLX and AMP to produce pharmaceutically functionalized choline monomers and their ^1^H–NMR spectra before anion exchange (**a**) and after modification to TMAMA/AMP (**b**) and TMAMA/CLX (**c**).

The proposed strategy for pharmaceutically functionalized copolymers included the modification of water-soluble ionic liquid, that is, [2-(methacryloyloxy)ethyl]trimethylammonium chloride (TMAMA/Cl), which contains a polymerizable methacrylate group and a chloride counterion (Cl^−^). The chloride ion was exchanged with pharmaceuticals anions, CLX^−^ and AMP^−^, derived from their sodium salts (NaCLX and NaAMP). This ion exchange reaction resulted in monomers carrying pharmaceutical anions, namely [2-(methacryloyloxy)ethyl]trimethylammonium cloxacillin (TMAMA/CLX) and ampicillin (TMAMA/AMP), as shown in Figure 1. The structures of the resulting monomers were confirmed by ^1^H-NMR spectroscopy to assess the efficiency of ion exchange by identifying characteristic proton signals of TMAMA. In particular, the trimethylammonium cation in the TMAMA unit was represented by 9 protons (signal F), and the pharmaceutical counterions (Figure 2). In the case of TMAMA/AMP the signal F at 3.16 ppm still existed after the exchange, but a new signal at 3.12 ppm appeared, indicating an exchange efficiency of 47% (Figure 2b). In contrast, for TMAMA/CLX, the signal slightly shifted to 3.14 ppm, proving a 100% anion exchange efficiency (Figure 2c). These results highlight the significant influence of cation–anion interactions, particularly the coordination number, on the efficiency of ion exchange. This reflects the ability of the cation to bind to the anion, as evidenced by the variations in exchange efficiency.

### 2.2. Linear Polymers Containing CLX^−^ as Single-Drug Delivery Systems 

**Figure 3:**
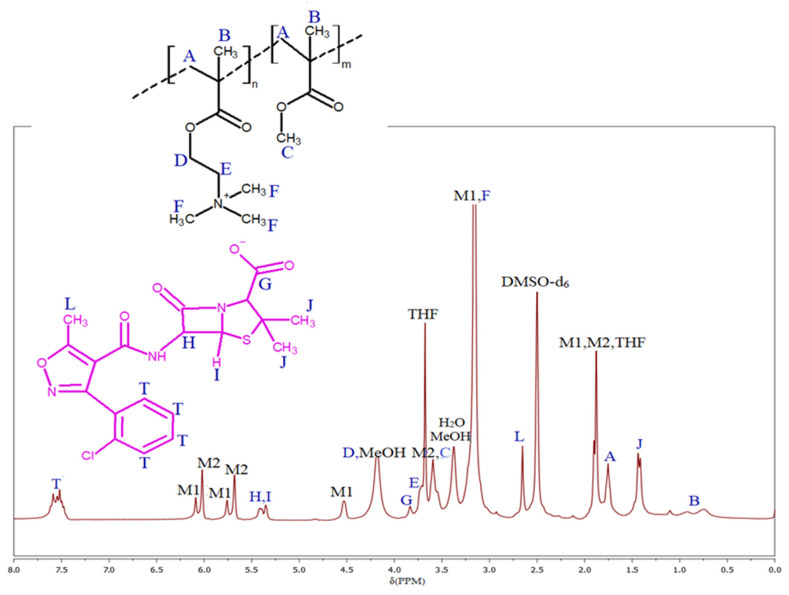
^1^H–NMR spectra of the reaction mixture at the end of polymerization in the synthesis of single-drug system IA (P(TMAMA/CLX–*co*–MMA)) (M1 and M2 are related to the signals of TMAMA/CLX and MMA, respectively).

The above-described ionic monomer TMAMA/CLX, with a choline species and a pharmaceutically active counterion, was copolymerized with the non-ionic monomer MMA in molar ratios of 25/75, 50/50, and 75/25 to synthesize well-defined linear copolymers, denoted as P(TMAMA/CLX–*co*–MMA)s (IA–IC), as single-drug systems carrying different ionic contents of TMAMA/CLX. For this purpose, the ATRP reactions were initiated by EBiB as a monofunctional initiator, catalyzed by CuBr/PMDETA complex, dissolved in MeOH/THF solvent mixture, and conducted at 40 °C (Figure 1). The conversion of the monomer to the polymer and resulting polymer structure was confirmed by ^1^H–NMR spectroscopy (Figure 3). The total conversion of both monomers (X) was calculated using the broad proton signal of the methyl group in the formed polymer backbone (B, 0.54–1.2 ppm) in relation to the signals from a proton in the vinyl groups of unreacted TMAMA and MMA monomers (identified as M1 and M2, in the range of 5.25 to 6.25 ppm). Moreover, the proton signal in the vinyl group of the unreacted monomer M1 at 6.09 ppm and the proton signal associated with the trimethylammonium group present in both the monomer and resulting polymer (referred to F at 3.02–3.30 ppm) were utilized to determine TMAMA conversion (X_M1_). Further, the conversion values were utilized to calculate additional parameters, including the degree of polymerization (DP), the ionic fraction content in the copolymer (F_M1_), and the average molecular weight of the copolymer (M_n_), as presented in Table 1. 

Linear copolymers were synthesized within a short time of 2 h, achieving total monomer conversions ranging from 40 to 75%, while the conversion of the ionic monomer remained similar (87–92%). The observed tendency of the monomer conversion to increase with the initial content of TMAMA monomer shows that the latter factor can be used to regulate the length of the polymer chain (DP_n_ = 160–299) and the number of ionic units (DP_M1_ = 87–277). The compositions of the resulting copolymers (IA–IC) revealed a higher ionic fraction content than anticipated based on the initial content of the ionic monomer (f_M1_/F_M1_: 25/54 (IA); 50/74 (IB); 75/93 (IC)). This phenomenon indicates higher reactivity of the ionic monomer (TMAMA/CLX), which is favored over MMA under polar conditions, probably leading to the formation of copolymers with gradient structures. 

### 2.3. Linear Polymers Containing CLX^−^/AMP^−^ as Dual-Drug Delivery Systems

**Figure 4:**
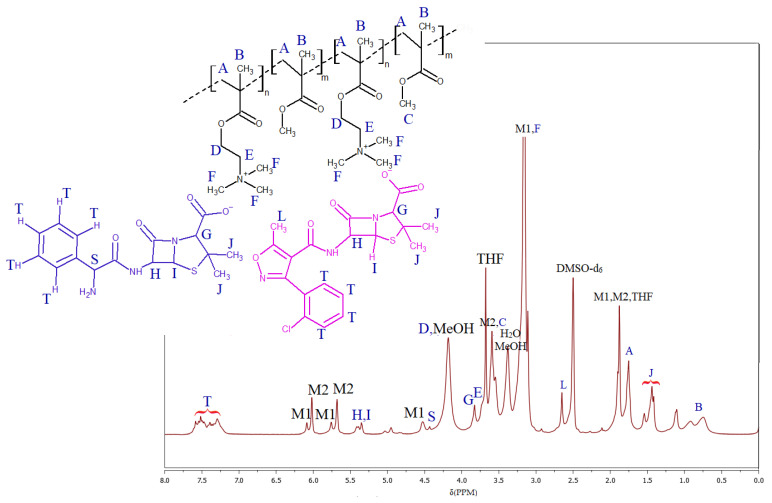
The ^1^H-NMR spectra of the reaction mixture at the end of the polymerization in the synthesis of dual-drug system IIA (P(TMAMA/CLX–*co*–MMA–*co*–TMAMA/AMP)) (M1 and M2 are related to the signals of TMAMA and MMA, respectively).

The dual-drug anions carried by the polymer conjugates were achieved through the terpolymerization of two pharmaceutically functionalized choline monomers, i.e., both TMAMA/CLX and TMAMA/AMP with MMA in ratios equal to 12.5:12.5:75, 25:25:50, and 37.5:37.5:25 via ATRP under conditions similar to those described above for the synthesis of single-drug copolymer conjugates (Figure 1). The resulting terpolymers, P(TMAMA/CLX–*co*–MMA–*co*–TMAMA/AMP) (IIA–IIC, Table 2), were designed to simultaneously transport two drugs. The conversion of monomers into polymers was evaluated using ^1^H–NMR spectroscopy, as shown in Figure 4, employing analogous signals such as those previously described for the CLX series. 

The presence of the second drug-based TMAMA in the polymerization with the lowest initial content of ionic monomers affected the formation of significantly higher chain lengths and similar numbers of incorporated ionic units when comparing IIA with IA (DP_n_/DP_M1_ = 239/91 vs. 160/87). This observation suggests differences in the relative reactivities of ionic comonomers, where TMAMA with the AMP counterion appears to be more reactive than TMAMA with the CLX counterion. The reduced reactivity of TMAMA/CLX is likely due to its higher steric hindrance, attributed to the Cl-substituted aromatic ring and extra five-membered ring. Furthermore, the structure of CLX renders it less hydrophilic than AMP, making TMAMA/AMP more favorable under the polymerization conditions employed. In relation to MMA, both TMAMAs polymerized faster, as evidenced by the correlation F_M1_ > f_M1_. In the system of three monomer components, MMA was converted at a higher rate than in the two-monomer system, leading to lower ionic content in the polymer IIA compared to IA (38% vs. 54%). However, a higher initial concentration of TMAMAs (f_M1_ = 50 and 75%) provided similar total monomer conversions, as well as DP_n_ and DP_M1_ values, to analogous systems in series I, but it required a longer duration of polymerization to be convenient for the incorporation of slower TMAMA/CLX. Generally, based on these relationships, the compositions of the resulting polymers IIA–IIC can be estimated from the initial proportions of TMAMA/MMA in the reaction mixture, which is related to the slightly higher content of ionic fraction in the polymer (f_M1_/F_M1_: 25/38 (IIA); 50/78 (IIB); 75/92 (IIC)). 

The SEC data were obtained for copolymers with an ionic fraction below 60%, which were very soluble in DMF. The molecular weight distribution for the dual-drug polymer IIA was larger compared to the single-drug polymer IA (Đ = 1.58 vs. 1.24), suggesting that the presence of two types of TMAMA counterions with differing natures promoted side reactions. It is worth noting that the SEC data may differ from the NMR-derived M_n_ values because the calibration standard used was PEO, a hydrophilic but non-ionic polymer, which differs in nature from the studied ionic copolymers.

**Figure 5:**
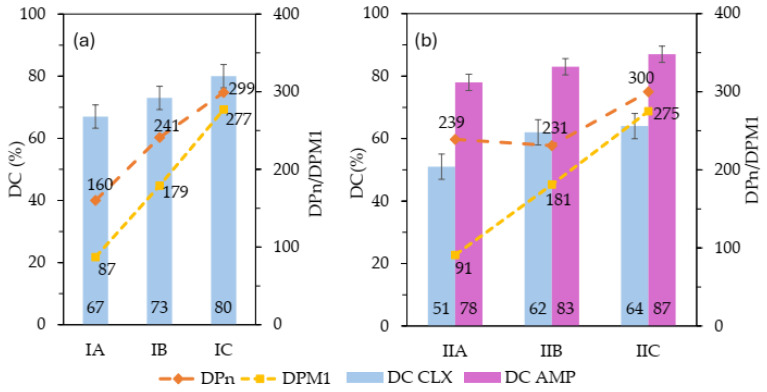
Correlation of drug content (DC) with polymer length chain (DP_n_) and number of TMAMA units (DP_M1_) in single (**a**) and dual (**b**) systems.

The drug content (DC), representing the percentage of pharmaceutical anions incorporated in the polymer chain, was determined by UV-Vis spectroscopy. The DC of CLX^−^ reached 67–80% in the single-drug systems (IA–IC) and 51–64% in the dual-drug systems (IIA–IIC), where the latter also co-delivered AMP^−^ with DC values of 78–87% (Figure 5). A simultaneous increase in DCs was observed with increasing total polymerization degree, including the degree of polymerization of TMAMA in the single-drug systems, as shown in Figure 5a (DC/DP_n_/DP_M1_: 67/160/87 (IA), 73/241/179 (IB), 80/299/277 (IC)). A similar trend of increasing DC was observed in the dual-drug systems, correlated with the number of TMAMA units, and with additional relationships between the DC of both drugs, that is, DC_CLX_ < DC_AMP_ (IIA: 51% CLX^−^ vs. 78% AMP^−^; IIB: 62% CLX^−^ vs. 83% AMP^−^; IIC: 64% CLX^−^ vs. 87% AMP^−^), as demonstrated in Figure 5b. In summary, the highest values for CLX^−^ were achieved for IC and IIC, with the longest polymer chain lengths and the highest ionic contents (DP_n_ ~ 300 and F_M1_ > 92%), which makes them the most advantageous systems.

### 2.4. Characteristics of Polymer Particles

**Figure 6:**
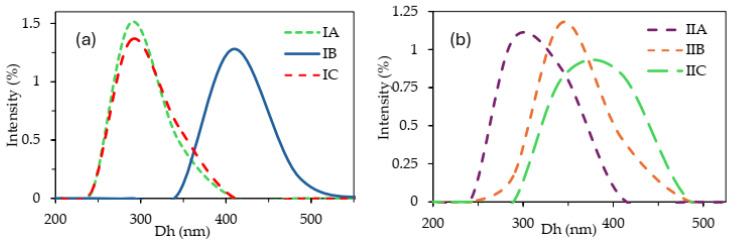
Particle size histograms of single (**a**)- and dual (**b**)-drug polymer systems obtained through DLS analysis. Dh represents hydrodynamic diameters.

Dynamic light scattering (DLS) analysis was conducted to assess the particle size of both types of drug delivery polymer conjugates in an aqueous solution (Table 3). The histograms in Figure 6 depict the particle size distributions, which exhibited a single fraction in all cases. The results for the single-drug polymer systems revealed distinct hydrodynamic diameters (Dh) for IA and IC of Dh ≤ 274 nm, whereas IB displayed a larger value of 380 nm. In the dual-drug systems (IIA–IIC), despite the differences in polymer structure, no significant influence was observed on particle sizes, which ranged from 288 nm to 348 nm. Furthermore, the polydispersity index (PDI), which defines the level of heterogeneity of particles as the distribution of their sizes, indicates uniformity of particle sizes (PDI ~0.01). Remarkably, slightly narrower size distributions were exhibited by IB and IIC (PDI: 0.006 and 0.008, respectively), which formed the biggest particles in both series. 

**Figure 7:**
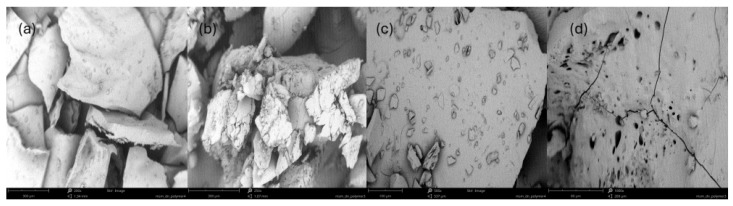
SEM for particles of single IC: 93 mol% of TMAMA (**a**,**c**) and IIA 38 mol% (**b**,**d**) at various magnitudes. (bar 200 nm (**a**,**b**); bar 100 nm (**c**,**d**)).

The polymer particles of different natures, single- vs. dual-drug systems, were monitored by SEM, showing slight differences in their morphologies depending on the composition and drug type. A brittle form of monolithic and smooth blocks was observed for copolymer IC containing CLX and a higher content of TMAMA (Figure 7a,b) than for copolymer IIA with the presence of accompanying AMP, providing a porous texture, which was crushable (Figure 7c,d).

### 2.5. Drug Release 

**Figure 8:**
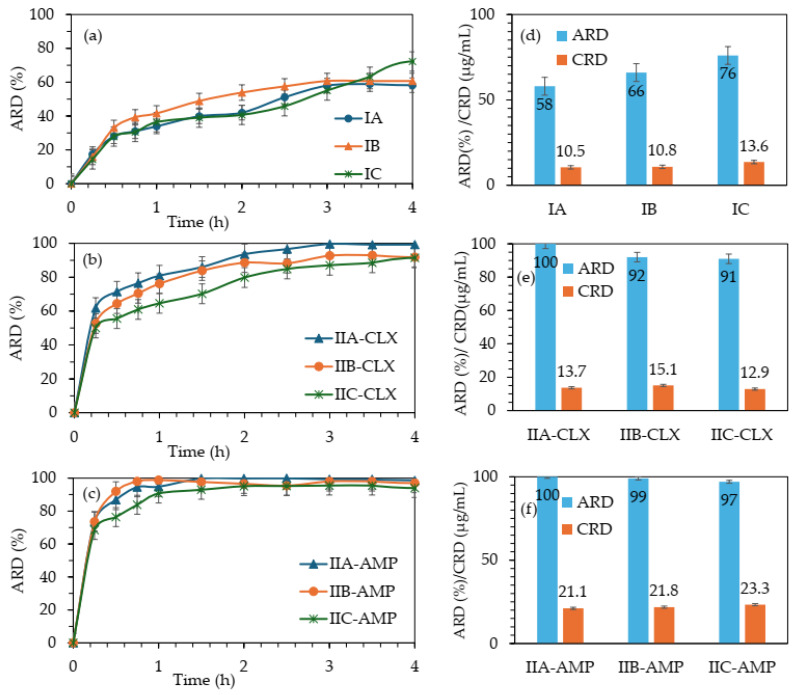
Kinetic release profiles for CLX^−^ in single-drug systems (**a**) and CLX^−^ and AMP^−^ in dual-drug systems (**b**,**c**) up to 4 h, and final ARD and CRD values after 72 h (**d**–**f**). ARD represents the amount of released drug, and CRD is the concentration of released drug.

In vitro drug release studies were conducted to monitor the exchange of CLX^−^ or CLX^−^/AMP^−^ anions in the polymer matrix by phosphate anions in the PBS using the dialysis method under physiological conditions in pH 7.4 at 37 °C for 72 h. Drug release from the polymer samples was detected by UV–Vis spectroscopy at specified time intervals as the percentage amount of released drug (ARD) and the concentration of released drug (CRD) (Figure 8). The kinetic profiles for the single-drug systems illustrated the release of 34–41% of CLX^−^ within 0.5–1 h, which increased to 58–72% within 4 h. The release stabilized over the next 74 h, with an additional 4–6% of drug release (Figure 8a). However, in the case of sample IC, the release profile differed from the others, exhibiting a more linear pattern. Initially, the release behavior was similar to system IA, but finally achieved the highest ARD value (Figure 8d). The increase in the amount of drug released from the single systems corresponded well with the elevated total polymerization degree and drug content (DP_n_/DC/ARD: 160/67/58 (IA), 241/73/66 (IB), 299/80/76 (IC)). Combining the effective content of the drug and its release, polymer IC appeared to be the most promising for achieving an extended therapeutic effect. 

In the context of the dual co-delivery systems, a significant initial burst release was observed in the first hour for both drugs, yielding 64–80% for CLX^−^ (Figure 8b) and 90–98% for AMP^−^ (Figure 8c). Following this, the release rate slowed considerably, with an additional 12–16% of CLX^−^ released over the next 2 h, ultimately reaching ARD values of 88–100% after 4 h. For systems with incomplete release, the process was extended up to 74 h, but slight progress was detected, maintaining a release plateau. In summary, complete release of both drugs was achieved by system IIA within 4 h, whereas the release from the IIB and IIC systems was slightly slower, reaching >90% of CLX^−^ and >97% of AMP^−^. The results for series II suggested that the interactions between CLX^−^ and AMP^−^ improved the co-release of CLX, although its DC was slightly lower than that in series I, but the polymers, especially IIA, were able to completely release both drugs in a relatively short time. 

The fitting to kinetic models (Figure S1, Table S1) indicated that CLX release follows the first-order kinetic model as the time depends on the percentage of remaining drug with high correlation coefficients (R^2^ = 0.87–0.98 for series I, and 0.91–0.99 for series II). Similar values of R^2^ were also obtained for data plotted from the Higuchi equation, which describes the percentage of drug release as a function of the square root of time, indicating the diffusion-controlled release of CLX (R^2^ = 0.91–0.97 for series I, and 0.9–0.98 for series II). The diffusion of the ionic CLX was also confirmed by the Korsmeyer–Peppas model, with R^2^ = 0.90–0.99, where the release exponent (n) in the equation M_t_/M = kt^n^ allowed us to define the release mechanisms. The systems in series I are on the border between a quasi-Fickian process (n ≤ 0.45) and a non-Fickian-type release as an anomalous diffusion process (0.45 < n < 0.89), while the latter was clearly exhibited by the systems in series II regardless of the drug release type. In the case of AMP release by double systems, the data fittings to all models were lower than for the release of CLX. They were characterized by the lowest R^2^ for the Higuchi model (0.65–0.78), whereas values elevated to 0.77–0.89 suggest a good fit, allowing the results to be described by first-order and Korsmeyer–Peppas equations.

## 3. Materials and Methods

### 3.1. Materials 

Methyl methacrylate (MMA, Alfa Aesar, Warsaw, Poland), tetrahydrofuran (THF, Sigma Aldrich, Poznan, Poland), and methanol (MeOH, Chempur, Piekary Śląskie, Poland) were dried using molecular sieves (type 4A, bulk density 640–670 kg/m^3^, Chempur, Piekary Śląskie, Poland) under an argon atmosphere. [2-(Methacryloyloxy)ethyl]trimethylammonium chloride (TMAMA/Cl, 80% aq. solution, Sigma-Aldrich, Poznan, Poland) was concentrated under vacuum conditions to obtain a solid product. Copper (I) bromide (CuBr, Fluka, Steinheim, Germany) was purified by stirring in glacial acetic acid, followed by filtration, washing with ethanol and diethyl ether, and subsequent vacuum drying. Deionized water was prepared using Hydrolab HLP Uv5 equipment (Straszyn, Poland). Other reagents used without prior purification included ethyl 2-bromoisobutyrate (EBiB), *N*,*N*,*N*′,*N*″,*N*″-pentamethyldiethylenetriamine (PMDETA), and ampicillin sodium salt (NaAMP) from Sigma Aldrich (Poznan, Poland) and cloxacillin sodium monohydrate (NaCLX) and deuterated dimethyl sulfoxide (DMSO–d6) from Alfa Aesar, as well as *N*,*N*-dimethylforamide (DMF, POCH, Gliwice, Poland), phosphate-buffered solution (PBS, Sigma Aldrich, Poznan, Poland), and diethyl ether (Chempur, Piekary Śląskie, Poland).

### 3.2. Preparation of Pharmaceutically Functionalized Choline Ionic Liquids Through Ion Exchange

The vacuum-dried TMAMA/Cl (2.14 mmol, 0.445 g) was dissolved in 2.2 mL of MeOH (forming solution 1). Next, NaCLX (2.14 mmol, 1.02 g) was dissolved in 5.1 mL of MeOH (TMAMA/Cl:MeOH = 1:5 *w*/*v*) and added dropwise to solution 1 with continuous stirring during the drug addition. Then, the mixture was stirred for 3 h constantly in a dark place at room temperature during the ion exchange reaction. After NaCl salt precipitation, the solution was filtered and washed twice with 1 mL MeOH to remove any salt. To accelerate MeOH evaporation, the filtrated solution containing TMAMA/CLX was left on a ventilator at room temperature for 30 min. It was further dried under vacuum conditions until it solidified into a powder product. Yield: 1.26 g. ^1^H NMR (DMSO–d_6_, 300 MHz, δ, ppm): 7.4–7.7 (4H, aromatic ring of CLX), 5.75 and 6.15 (2H, =CH_2_ in vinyl group), 5.25–5.45 (2H, -CH-N- and -CH-S- in β-lactam ring of CLX), 4.54 (2H, -CH_2_-O-), 3.82 (1H, -CH-N- in thiazolidine ring of CLX), 3.73 (2H, -CH_2_-N^+^-), 3.14 (9H, -N^+^(CH_3_)_3_), 2.65 (3H, -CH_3_ at isoxazole ring in CLX), 1.92 (3H, -CH_3_), 1.46 (6H, -C(CH_3_)_2_ in thiazolidine ring of CLX). Chloride content: 3.6%. 

The synthesis of analogical TMAMA/AMP followed the previously described method, utilizing equimolar ratios of TMAMA/Cl (3.9 mmol, 0.8 g) and NaAMP (3.9 mmol, 1.43 g) in MeOH (4 mL and 7.2 mL, respectively). However, the ion exchange process was completed within 20 h. Yield: 2.04 g. ^1^H–NMR (DMSO-d_6_, 300 MHz, δ, ppm): 7.1–7.5 (5H, -CH in aromatic ring in AMP), 5.75 and 6.15 (2H, -CH_2_ in vinyl group), 5.25–5.5 (2H, -CH-N- and -CH-S- in β-lactam ring of AMP), 4.54 (2H, -CH_2_-O-), 4.46 (1H, -CH-NH_2_ in AMP), 3.8–4.0 (1H, -CH-N- in thiazolidine ring of AMP), 3.75 (2H, -CH_2_-N^+^-), 3.16 and 3.12 (9H, -N^+^(CH_3_)_3_), 1.92 (3H, -CH_3_), 1.57 and 1.46 (6H, -C(CH_3_)_2_ in thiazolidine ring of AMP). Chloride content: 2.7%.

### 3.3. Synthesis of Copolymer Conjugates 

Ionic linear copolymers containing CLX anion, P(TMAMA/CLX–*co*–MMA) (example for IA): TMAMA/CLX (0.656 mmol, 0.41 g) was dissolved in 0.41 mL of MeOH in a Schlenk flask. Subsequently, MMA (1.968 mmol, 0.21 mL), THF (0.14 mL), and PMDETA (0.007 mmol, 0.001 mL) were added to the flask. The mixture was homogenized and degassed by performing two freeze–pump–thaw cycles. Then, EBiB (0.007 mmol, 0.001 mL) was introduced as the initiator, and an initial sample was taken. Another freeze–pump–thaw cycle was performed. Following this, a CuBr catalyst (0.007 mmol, 0.001 g) was quickly added to the mixture. The reaction flask was immersed in an oil bath at 40 °C. After 2 h, when a noticeable increase in the mixture’s viscosity was observed, the reaction was stopped by exposing the mixture to air. The resulting product was dissolved in MeOH (1 mL) and precipitated twice in a chloroform–diethyl ether mixture (1:1) to remove the catalyst from the polymer. The solvents were removed using a syringe, and the remaining solvents were evaporated under a ventilator at room temperature for 15 min. Finally, the polymer was dried under vacuum. Yield: 0.24 g. ^1^H–NMR (DMSO–d_6_, 300 MHz, δ, ppm): 7.4–7.7 (4H, aromatic ring of CLX), 5.3–5,5 (2H, -CH-N- and -CH-S- in β-lactam ring of CLX), 4.1–4.3 (2H, -CH_2-_O-), 3.82 (1H, -CH-N- in thiazolidine ring of CLX), 3.60–3.75 (2H, -CH_2_-N^+^-), 3.5–3.6 (3H, -O-CH_3_), 3.02–3.30 (9H, -N^+^(CH_3_)_3_), 2.65 (3H, -CH_3_ at isoxazole ring in CLX), 1.75 (2H, -CH_2_-C-), 1.36–1.46 (6H, -C(CH_3_)_2_ in thiazolidine ring of CLX), 0.54–1.2 (3H, -CH_3_). 

Dual bioactive ionic linear copolymers containing CLX^−^/AMP^−^, P(TMAMA/CLX–*co*–MMA–*co*–TMAMA/AMP) (example for IIA): TMAMA/CLX (0.557 mmol, 0.35 g) and TMAMA/AMP (0.557 mmol, 0.29 g) were dissolved in 0.64 mL of MeOH in a Schlenk flask. Then, MMA (3.342 mmol, 0.36 mL), THF (0.21 mL), and PMDETA (0.011 mmol, 0.002 mL) were added to the flask. Further steps were carried out, like for the synthesis and purification of P(TMAMA/CLX–*co*–MMA), using proper amounts of EBiB (0.011 mmol, 0.002 mL) and CuBr (0.011 mmol, 0.002 g). Yield: 0.73 g. ^1^H–NMR (DMSO–d_6_, 300 MHz, δ, ppm): 7.1–7.7 (9H, -CH in aromatic rings of AMP and CLX), 5.25–5.4 (2H, -CH-NH- and -CH-S- in β-lactam ring of CLX and AMP), 4.43 (1H, -CH-NH_2_ in AMP), 4.1–4.3 (2H, -CH_2-_O-), 3.75–3.87 (2H, -CH-N- in thiazolidine ring of CLX and AMP), 3.65–3.75 (2H, -CH_2_-N^+^-), 3.4–3.65 (3H, -O-CH_3_), 3.05–3.25 (9H, -N^+^(CH_3_)_3_), 2.65 (3H, -CH_3_ at isoxazole ring in CLX), 1.75 (2H, -CH_2_-C), 1.4–1.7 (12H, -C(CH_3_)_2_ in thiazolidine ring of CLX and AMP), 0.6–1.2 (3H, -CH_3_).

### 3.4. Release Studies of Copolymer Conjugates Containing CLX^−^ and AMP^−^

Copolymer conjugates containing drug anions (1 mg) were dissolved in 1 mL of PBS (pH 7.4) to prepare a 1 mg/mL solution. The solution was then transferred into a dialysis cellulose membrane bag with a molecular weight cutoff (MWCO) of 3.5 kDa. Then, this membrane bag was placed inside a glass vial filled with 44 mL of PBS and stirred at 37 °C during a 74 h dialysis process. The release progress was monitored by assessing changes in drug concentration in the external PBS solution surrounding the dialysis bag. At different time intervals, 0.5 mL samples of the solution containing the released drug were collected, and 0.5 mL of MeOH was added to the cuvette. These samples were subsequently analyzed using a UV-Vis spectrophotometer to quantify the released drug amount by measuring the absorbance at a wavelength of λ = 206 nm for AMP^−^ and λ = 203 nm for CLX^−^. Each reported result represents the average of three measurements.

### 3.5. Characterization Methods

Proton nuclear magnetic resonance (^1^H–NMR) spectra were acquired using a UNITY/NOVA spectrometer (Varian, Mulgrave, Victoria, Australia) operating at a frequency of 300 MHz. Measurements were performed on samples dissolved in DMSO–d_6_ with tetramethylsilane used as the internal standard. Size exclusion chromatography (SEC) was conducted using an Ultimate 3000 chromatograph (Thermo Fisher Scientific, Waltham, MA, USA) equipped with a RefractoMax 521 differential refractometer detector. Polymer samples prepared in DMF containing 10 mM LiBr at 40 °C, were passed through a precolumn TSKgel Guardian SuperMP(HZ)-H (4.6 mm × 2 cm, with a particle size of 6 μm), followed by two columns of TSKgel SuperMilipore HZ-H (4.6 mm × 15 cm, with a particle size of 6 μm). The flow rate was maintained at 0.45 mL/min. The average molecular weight (M_n_) and dispersity index (Ð) were determined on the basis of poly(ethylene oxide)/poly(ethylene glycol) (PEO) standards with molecular weights ranging from 982 to 969,000 g/mol. The drug content (DC) and the amount of released drug (ARD) were analyzed by ultraviolet–visible spectroscopy (UV-Vis) using an Evolution 300 spectrometer (Thermo Fisher Scientific, Waltham, MA, USA). Polymer samples dissolved in a PBS and MeOH (1:1) at a concentration of 0.05 mg/mL were placed in a quartz cuvette and measured at a maximum wavelength of λ = 206 nm for AMP^−^ and λ = 203 nm for CLX^−^. Calibration curves were prepared for drug concentrations ranging from 0.1 mg/mL to 0.006 mg/mL in a PBS-MeOH mixture (1:1). Dynamic light scattering (DLS) was performed using a Nanotrac Flex with a Mictrac MRB laser particle size analyzer (Microtrac Retsch GmbH, Haan, Germany; Dimensions LS software 1.1.0.), equipped with an external “dip-in” probe with 180° backscattering. Polymer samples were dissolved in deionized water at a concentration of 1.0 mg/mL to determine the hydrodynamic diameters (Dh) and polydispersity index (PDI). Each sample was measured at least three times to ensure reproducibility. The morphology of the polymers was observed by scanning electron microscopy (SEM) using a Phenom ProX electron microscope (PhenomWorld, Eidhoven, The Netherlands) operating at 15 kV. Before measurements, the sample was dusted with a layer of 5 nm gold nanoparticles. 

## 4. Conclusions

The well-defined linear copolymers, including series I with CLX^−^ (single-drug DDS) and series II containing both CLX^−^ and AMP^−^ (dual-drug DDS), were synthesized to demonstrate their potential as nanocarriers (274–380 nm (CLX^−^) and 288–348 nm (CLX^−^/AMP^−^)). In both series, the TMAMA monomers functionalized by pharmaceutical anions with antibiotic activities were incorporated into the polymer chains at different contents (38–93 mol%). The various lengths of the polymer chains (DP_n_ = 160–300) were adjusted through monomer conversion (40–75%), while the initial proportion of comonomers allowed for the regulation of ionic contents (38–93 mol%) within the polymers. 

The drug content within the polymer matrix with 67–80% in the CLX^−^ systems, and 51–64% of CLX^−^ and 78–87% of AMP^−^ in the dual-drug systems, corresponded to the efficient in vitro release of 91–100% of CLX^−^ (12.9–15.1 µg/mL) and 97–100% of AMP^−^ (21.1–23.3 µg/mL) from the dual-drug systems, and 67–80% of CLX^−^ in the single-drug systems, in PBS within 72 h, but AMP release was almost complete after 4 h. The drug content and release were significantly affected by structural factors, such as the polymer chain length, ionic fraction content in the copolymer, and pharmaceutical anion’s nature. Overall, the linear copolymers proved effective in designing both single- and dual-drug systems with tailored release profiles to facilitate efficient drug delivery. The cholinium-based polymer systems combining AMP^−^ and CLX^−^ demonstrated significant potential for antibiotic treatment, enabling the simultaneous co-delivery of drugs for optimized therapeutic outcomes.

## Figures and Tables

**Table 1:** Characteristics of linear copolymer P(TMAMA/CLX–*co*–MMA) synthesized by ATRP.

No.	f_M1_/f_M2_ (mol%)	Time (h)	X_M1_^a^ (%)	X ^a^ (%)	DP_M1_^a^	DP_n_^a^	F_M1_^a^ (mol)	M_n_^a ^ (g/mol)	M_n_^b^ (g/mol)	Ð ^b^
**IA**	25/75	2	87	40	87	160	0.54	59,900	10,700	1.24
**IB**	50/50		90	60	179	241	0.74	115,100	-	-
**IC**	75/25		92	75	277	299	0.93	170,200	-	-

M1 = TMAMA/CLX, M2 = MMA; conditions: [TMAMA/CLX + MMA]_0_:[EBiB]_0_:[CuBr]_0_:[PMDETA]_0_ = 400:1:1:1; MeOH:TMAMA/CLX = 1:1 υ/ωt, MeOH:THF = 3:1 υ/υ, 40 °C; f_M1_, f_M2_—initial content of monomer in reaction mixture; X_M1_^a^—TMAMA conversion; X—total monomer conversion; DP_M1_—polymerization degree of ionic monomers; DP_n_—total polymerization degree; F_M1_—content of ionic fraction in polymer; M_n_—average molecular weight; Đ—dispersity index. ^a^ determined by ^1^H-NMR (DMSO-d6); ^b^ determined by SEC (DMF, PEO calibration); - means not determined.

**Table 2:** Characteristics of linear terpolymers P(TMAMA/CLX–*co*–MMA–*co*–TMAMA/AMP) synthesized by ATRP.

No.	f_M1_/f_M2_ (mol%)	Time (h)	X_M1_^a^ (%)	X ^a^ (%)	DP_M1_^a^	DP_n_^a^	F_M1_^a^ (mol)	M_n_^a ^ (g/mol)	M_n_^b^ (g/mol)	Ð ^b^
**IIA**	25/75	2	91	60	91	239	0.38	55,500	16,200	1.58
**IIB**	50/50	3.5	90	58	181	231	0.78	85,400	-	-
**IIC**	75/25	3	92	75	275	300	0.92	124,800	-	-

M1 = TMAMA/CLX + TMAMA/AMP, M2 = MMA; cond.: [(TMAMA/CLX + TMAMA/AMP) + MMA]_0_: [EBiB]_0_: [CuBr]_0_: [PMDETA]_0_ = 400:1:1:1; MeOH:TMAMA = 1:1 υ/ωt, MeOH:THF = 3:1 υ/υ, 40 °C; f_M1_, f_M2_—initial content of comonomers in reaction mixture; X_M1_^a^—conversion of both TMAMA monomers; X—total conversion; DP_M1_—polymerization degree of ionic monomers; DP_n_—total polymerization degree; F_M1_—content of ionic fraction in polymer; M_n_—average molecular weight of copolymer; Đ—dispersity index. ^a^ determined by ^1^H-NMR (DMSO-d6), ^b^ determined by SEC (DMF, PEO calibration); - means not determined.

**Table 3:** DLS characteristics of linear polymer particles carrying drug.

Type DDS	Polymer	Dh (nm)	PDI
Single	IA	274	0.01
	IB	380	0.006
	IC	277	0.01
Dual	IIA	288	0.01
	IIB	324	0.01
	IIC	348	0.008

Here, Dh is hydrodynamic diameter and PDI is polydispersity index.

## Data Availability

All data generated or analyzed during this study are included in this published article.

## Supplementary Materials

The following supporting information can be downloaded at: https://www.mdpi.com/article/10.3390/molecules29245973/s1, Figure S1: Fitting of kinetic release profiles for single- and dual-drug systems to first-order (a–c), Higuchi (d–f), and Korsmeyer–Peppas (g–i) models. Table S1: Correlation coefficients (R^2^) for kinetic model equations and drug release exponents (n).
