# Supplementary material for: Polymerizable Cholinium-Based Antibiotics for Polymer Carriers: Systems with Combined Load of Cloxacillin and Ampicillin
Source: Molecules. 2024 Dec 18;29(24):5973. doi: 10.3390/molecules29245973 (PMC11678903; doi:10.3390/molecules29245973)
Supplement: Supplementary file 1 [file molecules-29-05973-s001.zip › molecules-3358333-supplementary.pdf]

Supplementary Materials

Polymerizable Cholinium-Based Antibiotics for Polymer Carriers: Systems with Combined Load of Cloxacillin and Ampicillin

Content:

Figure S1. Fitting of kinetic release profiles for single and dual drug systems to the first-order (a-c), Higuchi (d-f), and Korsmeyer-Peppas (g-i) models.

Table S1. Correlation coefficients (R^2) for kinetic model equations and drug release exponents (n).

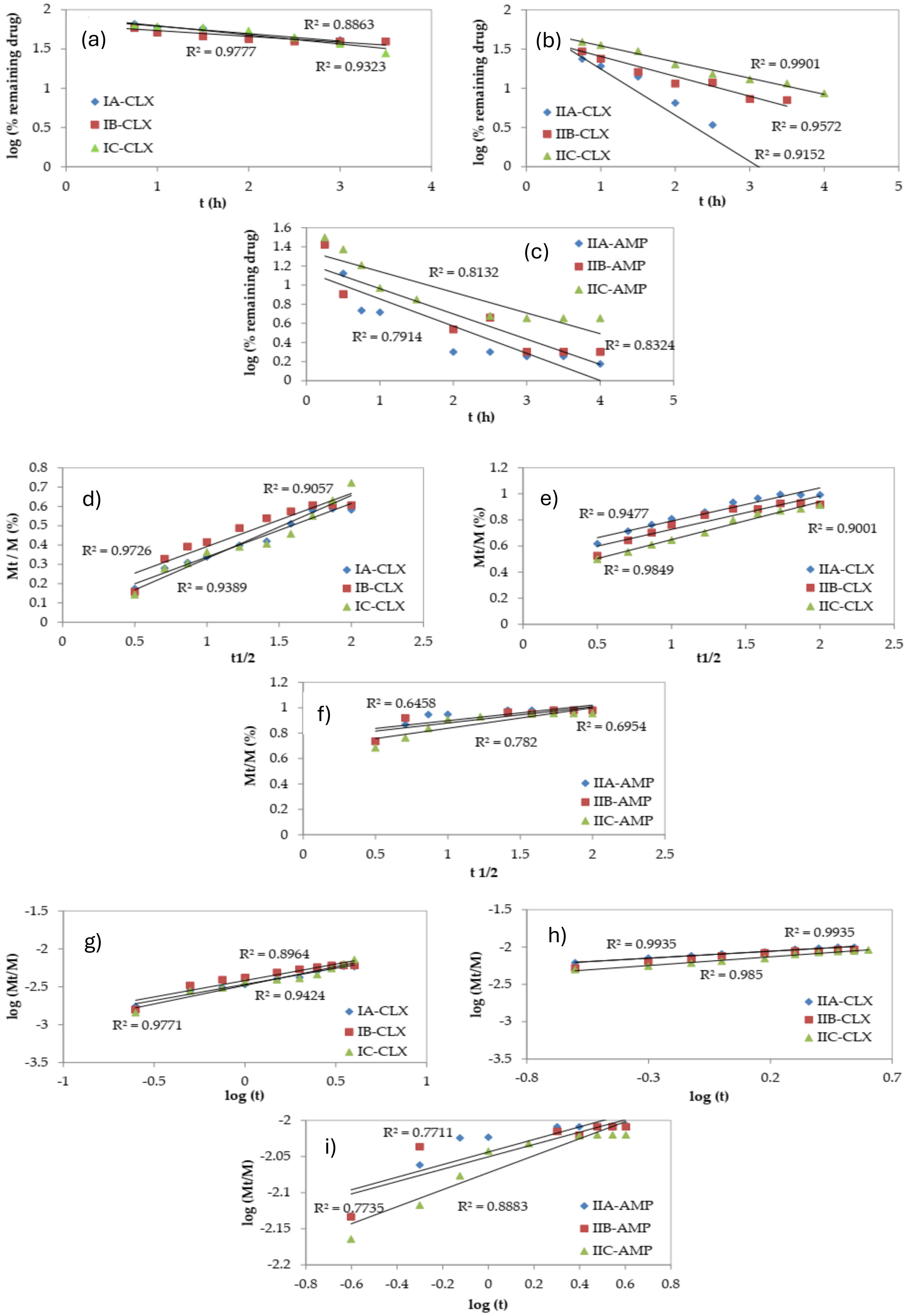

Figure S1. Fitting of kinetic release profiles for single and dual drug systems to the first-order (a-c), Higuchi (d-f), and Korsmeyer-Peppas (g-i) models.

Table S1. Correlation coefficients (R^2) for kinetic model equations and drug release exponents (n).

Type	Drug	Polymer	First-Order	Higuchi	Korsmeyer-Peppas	
			R^2	R^2	R^2	*n*
**Single**	CLX^-	**IA**	0.9777	0.9726	0.9771	0.43
		**IB**	0.8863	0.9057	0.8964	0.43
		**IC**	0.9323	0.9389	0.9424	0.49
**Dual**	CLX^-	**IIA**	0.9152	0.9477	0.9935	0.19
		**IIB**	0.9572	0.9001	0.9935	0.20
		**IIC**	0.9901	0.9849	0.9850	0.18
	AMP^-	**IIA**	0.7914	0.6458	0.7711	0.09
		**IIB**	0.8324	0.6954	0.7735	0.09
		**IIC**	0.8132	0.7820	0.8883	0.12
